# Transthyretin Amyloid Cardiomyopathy Treatment: An Updated Review

**DOI:** 10.3390/jcm14176089

**Published:** 2025-08-28

**Authors:** Dinusha Wanniarachchige, Shazli Khan, Stephen Pan

**Affiliations:** 1 School of Medicine, New York Medical College, Valhalla, NY 10595, USA; dwanniar@student.nymc.edu; 2 Division of Cardiology, Columbia University Irvington Medical Center, New York, NY 10032, USA; 3 Infiltrative Cardiomyopathy Program, Department of Cardiology, Westchester Medical Center, Valhalla, NY 10595, USA

**Keywords:** transthyretin amyloidosis, cardiomyopathy, heart failure, TTR stabilizer, TTR silencing, TTR scavenger

## Abstract

Transthyretin amyloid cardiomyopathy (ATTR-CM) is a progressive form of restrictive cardiomyopathy caused by the misfolding and deposition of transthyretin protein. What was once a condition that had little by way of treatment other than liver transplantation has now become a manageable disease due to revolutionary novel therapies. This review outlines therapeutic strategies aimed at halting or reversing disease progression. Current approaches include transthyretin (TTR) stabilizers such as tafamidis and acoramidis, as well as TTR silencers such as vutisiran. In addition, novel therapies under clinical investigation are emerging, such as gene-editing strategies and monoclonal antibodies targeting amyloid deposits. The aim of this paper is to investigate the current literature and ongoing clinical trials exploring treatment options for ATTR-CM. Transthyretin stabilizers and RNA silencers improve survival, functional capacity, and quality of life. Early-phase studies of gene-editing and monoclonal antibody therapies demonstrate promising amyloid-lowering effects. Stabilizers and silencers continue to constitute the standard therapy, while gene-editing and monoclonal antibodies offer potential as future treatments. The advent of a multitude of therapies for ATTR-CM holds promise for a future of targeted, personalized medicine for the treatment of this not-so-uncommon cause of heart failure.

## 1. Introduction

Transthyretin (TTR) is a transport protein synthesized by the liver that binds thyroxine, the retinol/retinol binding protein (RBP) complex, and other substances. TTR is also known as prealbumin, as it was first discovered as the protein that migrated in front of albumin in an electrophoresis gel. However, it is unrelated to albumin, as they have different amino acid sequences [1].

It is a 127-amino acid 55 kD protein. Small amounts are produced by the choroid plexus and the retina, amongst other tissues, but the main source of systemic TTR is the liver. It exists in vivo primarily as a tetramer of identical subunits; it rarely exists as a monomer. It is used clinically as a marker for nutritional status, as levels can be sensitive to protein intake. It also serves as a marker for inflammation as a negative acute-phase reactant.

If the tetrameric protein dissociates into monomers, it becomes susceptible to misfolding and accumulation into soluble oligomers, protofilaments, and fibrils [2]. These fibrils can then deposit in various organ tissues, causing a subtype of the protein-deposition disease termed amyloidosis.

TTR amyloidosis can have many clinical manifestations, including, but not limited to, peripheral and autonomic neuropathy, orthopedic complaints (i.e., carpal tunnel disease, trigger finger, spinal stenosis, and tendon ruptures), and gastrointestinal dysomotility. However, one of the most morbid manifestations of the disease is cardiac deposition causing a restrictive cardiomyopathy known as transthyretin amyloid cardiomyopathy, or ATTR-CM [2,3].

This condition can either result from a mutation in the *TTR* gene that is inherited in an autosomal dominant pattern (ATTRm) or occur by deposition of an unaltered/wildtype TTR protein that is associated with advanced age (ATTRwt). It is a condition that predominantly affects men at least 60 years of age, and largely has a poor prognosis once diagnosed, with median survival being 2.5 and 3.6 years for ATTRm and ATTRwt, respectively. ATTRwt generally manifests with cardiac and orthopedic complaints, and the diagnosis is often missed for years. This delay in diagnosis is often attributable to early clinical manifestations being mistaken for age-related changes. In contrast, ATTRm presents more commonly with neuropathy, although the clinical manifestations can vary depending on the genotype. The V122I (also known as the V142I) variant is the most common pathological TTR variant in the United States. It can be found in up to 3.4% of all African Americans in genetic epidemiology studies, although penetrance is age-related and incomplete [3]. Nonetheless ATTR-CM remains significantly underdiagnosed, with prevalence estimates of 10–20% in patients over the age of 65 with left ventricular hypertrophy or heart failure with preserved ejection fraction [4,5].

## 2. Clinical Manifestations and Diagnosis

One of the major limitations associated with the delivery of appropriate and timely treatment is delayed diagnosis. Patients’ comorbidities and often non-specific presenting symptoms, especially in earlier stages of ATTR-CM disease, likely explain this delay. Common initial misdiagnoses include hypertensive cardiomyopathy, hypertrophic cardiomyopathy, ischemic heart disease, aortic stenosis, and heart failure with preserved ejection fraction (HFpEF) [6]. ATTR-CM should be high on a differential diagnosis list if a patient with left ventricle wall thickening is seen in combination with one or more additional factors classified as “red flags.” Yamamoto and Yokochi organized these “red flags” into demographics, family history, clinical history, clinical exam, imaging, and alert signs. These included patients over the age of 60, those with a family history of progressive neuropathy or heart failure at an early age, HFpEF in the absence of hypertension or low-flow aortic stenosis, low voltage or conduction abnormalities on ECG, and intolerance to heart-failure drugs [7]. The vast range of clinical symptoms and exam/imaging findings emphasizes the importance for physicians that ATTR-CM be kept high on the list of differential diagnosis [8].

## 3. Current Disease-Directed Therapies

**Figure 1:**
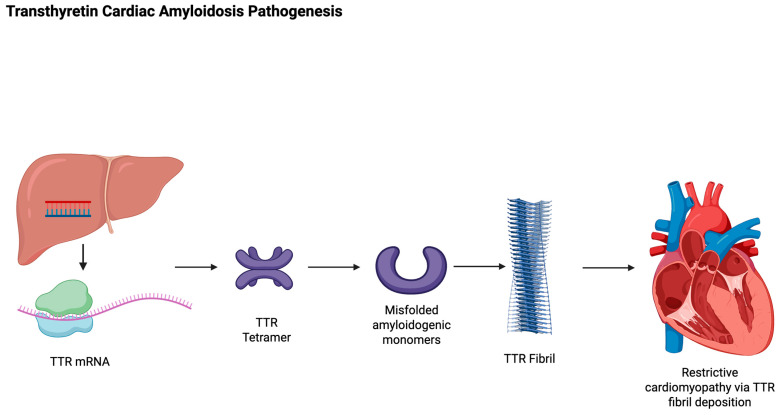
Schematic of the pathogenesis of TTR amyloid heart disease. Wanniarachchige, D. Created in BioRender. 2025. Available online: https://BioRender.com/t4xt235 (accessed on 19 August 2025).

**Figure 2:**
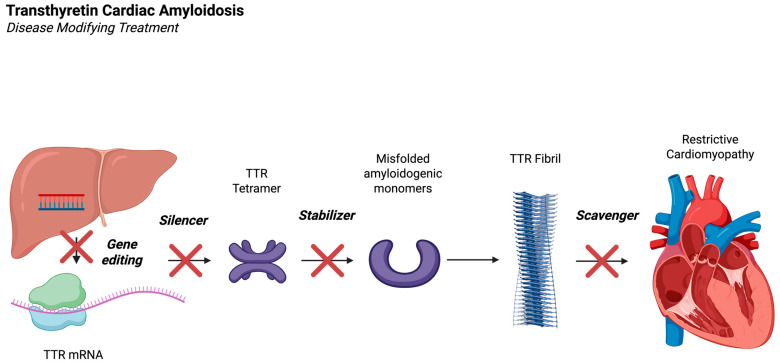
Schematic of the mechanism of action of current disease-modifying treatments for TTR amyloid disease. Wanniarachchige, D. Created in BioRender. 2025. Available online: https://BioRender.com/hpj4fos (accessed on 19 August 2025).

The treatment of ATTR-CM includes addressing the cardiac symptoms of the disease as well as treatment of the underlying pathology. Figure 1 outlines the pathogenesis of ATTR-CM. The etiology of ATTR-CM comprises poor stability and subsequent depolymerization of the TTR tetramer into monomers. Aggregates of these monomers create the oligomers, protofilaments, and amyloid fibrils, which deposit in multiple organs [2]. The historically high mortality in ATTR-CM has been largely attributable to prior standards of care that solely addressed symptomatic management. In fact, for decades, the only available treatment to stop disease progression was liver transplantation, in those with ATTRv [9]. However, the availability of transplant organs, as well as various comorbidities and the advanced age of the patient population, posed a rather large limitation. Fortunately, this methodology of treatment has largely been replaced with novel therapies aimed at either (1) **stabilizing** the TTR tetramer, or (2) **silencing** the production of TTR by the liver, or (3) **scavenging** or removing TTR amyloid deposits within the body (Figure 2). While many of these potential therapies remain under study, TTR stabilizers and silencers are both currently available in the USA for use in those with this disease, with expanding indications.

### 3.1. Transthyretin Stabilizers

#### 3.1.1. Tafamidis

Tafamidis became the first drug therapy specifically approved for ATTR-CM with its approval in 2019. This molecule is a derivative of a benzoxazole that was selected for its lack of nonsteroidal anti-inflammatory drug activity [10]. It was developed in the laboratory of Jeffrey Kelly at Scripps Research in San Diego, California, specifically for the purpose of stabilizing the TTR tetramer by binding unoccupied thyroxine binding sites [10]. In doing so, it effectively prevents monomer disassociation and later re-aggregation into the fibrils that cause disease [Figure 2]. Tafamidis was originally approved for the treatment of polyneuropathy in Europe and was later investigated for its utility in cardiomyopathy in the ATTR-ACT trial published in 2018 [11]. This study was a multicenter, international, parallel-design, placebo-controlled, double-blind, randomized trial that randomized 441 patients with ATTR-CM, diagnosed mostly by biopsy (PYP scan was allowed as a diagnostic pathway as a protocol change later in the trial), with New York Heart Association (NYHA) class I-III symptoms. In all, 264 patients were assigned to the tafamidis treatment group and 177 patients assigned to placebo. The treatment group was further stratified with half of the tafamidis patients receiving a 20 mg dose and the other half receiving 80 mg daily [2]. Primary outcomes were all-cause mortality and frequency of cardiovascular-related hospitalizations over a 30-month period. Secondary outcomes were the change from baseline in the 6 min walk test and the Kansas City Cardiomyopathy Questionnaire—Overall Summary (KCCQ-OS) score, a measure of functional capacity and quality of life, respectively [2].

The ATTR-ACT trial demonstrated that tafamidis significantly improved outcomes in patients with ATTR-CM, compared to placebo. Tafamidis reduced all-cause mortality by 30% and cardiovascular-related hospitalizations in those who survived the trial by approximately 30% at the 30-month follow-up [2]. The trial also highlighted the danger of not treating patients with this disease: the placebo group had a shockingly high 42.9% mortality rate at the 30-month mark. The reduction in mortality was quite significant, both clinically and statistically, with a hazard ratio of 0.70 (95% CI: 0.51–0.96). The number needed to treat in the trial relative to the reduction in mortality was an impressive 7.5.

Secondary outcomes showed that tafamidis significantly slowed the decline in functional capacity, as measured by the 6 min walk test, and reduced the decline in quality of life, as assessed by the KCCQ-OS score. Exploratory findings included smaller increases in N-terminal pro-B-type natriuretic peptide (NT-proBNP, a marker for volume overload) levels at months 12 and 30, and a smaller decline in left ventricular stroke volume over 30 months. Subgroup analyses confirmed consistent benefits across most categories, although patients with NYHA class III disease had higher hospitalization rates in the tafamidis group, which was attributed to longer survival despite severe disease stages [2]. Tafamidis and placebo groups exhibited similar safety profiles, with generally mild adverse events and few tafamidis patients discontinuing due to adverse events. Importantly, the mortality benefit was only noted after ~18 months of therapy, highlighting the importance of earlier diagnosis and treatment. Together, these findings demonstrate tafamidis to be an effective therapy for reducing mortality, hospitalizations, and functional and quality-of-life declines in this patient population [2].

#### 3.1.2. Diflunisal

Diflunisal is a classic nonsteroidal anti-inflammatory drug (NSAID) that also has high binding affinity for the TTR tetramer, allowing it to also be utilized as a TTR stabilizer [12]. With any NSAID use, monitoring renal function is essential, as a decline in renal function is a known adverse effect [13]. As a generic drug, diflunisal has been suggested as a cost-effective alternative off-label treatment for patients with ATTR-CM. A retrospective cohort study at the Boston University Amyloidosis Center demonstrated that diflunisal treatment was associated with improved survival in patients with early-stage ATTRwt [14]. The study determined that diflunisal was effective in reducing mortality in ATTRwt patients, based on baseline cardiac biomarkers, renal function, left ventricular (LV) ejection fraction, and septal thickening, after a 4-year follow-up [14]. Comparisons between diflunisal and tafamidis were made based on cost and effectiveness. Diflunisal is a generic NSAID for which some evidence suggests that it can reduce mortality in ATTRwt; however, it is associated with renal and GI side effects, making medication regimen compliance difficult, and prompting persistent concerns about the safety of long-term use [14]. Thus, in the treatment of ATTR-CM, diflunisal remains a consideration only when other therapies are unavailable.

#### 3.1.3. Acoramidis

There are many documented variants of the TTR gene that have been associated with disease, with varying clinical manifestations and prognoses. The aforementioned V122I (V142I) variant generally presents with cardiomyopathy at an older age, whereas the V30M (V50M) variant typically presents with neuropathy, or initially with milder cardiomyopathy development later in life. Just as disease manifestations can vary between variants, so too can the effect on TTR stability, but in general, most disease-associated variants have been found to decrease the stability of the TTR tetramer, leading to the accumulation of misfolded amyloid fibrils. On the other end of the disease spectrum, Hammarström et al. found that a T119M variant in the TTR gene exhibited improved stabilization of the TTR tetramer compared to wildtype [15]. Further work showed that this T119M variant actually was protective in individuals that were also carriers of the pathogenic V30M gene variant, preventing or delaying the development of clinically evident amyloid disease [16].

Acoramidis is a TTR stabilizer that endorses near-complete stabilization of the TTR tetramer in both ATTRwt and ATTRm [16]. The efficacy of acoramidis comes from mimicking the protective T119M variant. In vitro, acoramidis has been shown to have more favorable binding thermodynamics, binding affinity, binding-site occupancy, and potency, compared to both tafamidis and diflunasil [17]. Gillmore et al. conducted a phase 3, double-blind trial (ATTRibute) similar in design to the tafamidis phase 3 trial conducted by Maurer et al. The ATTRibute trial was a phase 3 trial evaluating the efficacy of acoramidis in transthyretin amyloid cardiomyopathy. In all, 632 patients aged 18–90 years with a confirmed diagnosis of the condition were randomized in a 2:1 ratio to receive acoramidis hydrochloride (800 mg twice daily) or placebo for 30 months [16]. The acoramidis intervention group demonstrated 80.7% survival at 30 months, which was a significant improvement compared to the 74.3% survival rate in the control group. Regarding morbidity- or cardiovascular-related hospitalizations, the treatment group had 0.29 hospitalizations per year, compared to 0.58 in the control group. This translated to a relative risk reduction of 50.4%. Furthermore, significant clinical benefits compared to placebo were seen in NT-proBNP levels at 30 months [16]. Similar to treatment with tafamidis, use of acoramidis resulted in improvements in quality of life, as measured by the 6 min walk test and the KCCQ-OS score. Serum TTR levels were consistently higher in the acoramidis group, with a mean difference of 7.01 mg/dL, highlighting the ability of acoramidis to reach near-complete stabilization of TTR. Although mortality rates were not significantly different, the acoramidis group experienced fewer cardiovascular-related hospitalizations and serious adverse events, compared to the placebo group [16].

There were many key differences to note between the ATTRibute and ATTR-ACT trial. Upon direct comparison, mortality, in both the placebo and intervention arms, was significantly lower in ATTRibute as compared to ATTR-ACT. Furthermore, when considering the endpoint of mortality alone, the ATTRibute trial did not demonstrate a statistically significant benefit. This likely relates, at least in part, to improved survival in the contemporary ATTR-CM patient population due to improved recognition and earlier diagnosis, a factor which made it more challenging to demonstrate mortality differences in the short term. In addition, the ATTRibute trial had a higher proportion of patients with ATTRwt, which has a slower disease progression and lower short-term mortality compared to ATTRv.

In the ATTR-ACT trial, survival curves began to diverge at 18 months. In the ATTRibute trial, more patients had early-stage ATTR-CM, as evidenced by greater proportion of patients in earlier NYHA disease classes (I and II). That, combined with the fact that patients in the intervention group were allowed to take tafamidis, likely lowered short-term mortality. In sum, 17.5% of patients in the ATTRibute trial eventually received tafamidis, which may have improved outcomes overall and reduced any detectable mortality difference [16]. Lastly, from a statistical standpoint, ATTRibute was not powered for mortality as a primary endpoint, while the ATTR-ACT trial was.

Biochemically, both acoramidis and tafamidis function as TTR stabilizers, but their binding characteristics differ significantly. Tafamidis binds to the thyroxine-binding sites of the TTR tetramer, predominantly through hydrophobic interactions, stabilizing the tetrameric structure and preventing dissociation into the monomers that lead to amyloid fibril formation [2]. Acoramidis mimics the natural stabilizing effect of the T119M TTR variant, which uses hydrogen bonding to confer exceptional thermodynamic stability [16]. This enthalpy-driven binding mechanism allows acoramidis to achieve over 90% TTR stabilization across all tested variants in an in vitro model, a level that surpassed tafamidis. These differences in binding affinity and thermodynamics suggest that acoramidis may provide enhanced biochemical stability relative to TTR. It has also been noted that acoramidis does not cross the blood–brain barrier, whereas tafamidis does. Whether these differences impact clinical outcomes remains to be seen. It should especially be noted that in ATTRibute, patients on both acoramidis and tafamidis therapies simultaneously showed no increased benefit as compared to those on tafamidis alone, which likely is a result of the similar mechanisms of action.

Based on the results of the ATTRibute trial, the FDA approved the clinical use of acoramidis for the treatment of TTR cardiomyopathy (both wildtype and variant) to reduce cardiovascular death and hospitalizations, in late November 2024.

### 3.2. TTR Gene Silencing Therapies

TTR-silencing agents are an emerging class of agents that target the TTR mRNA [Figure 2]. This can be accomplished using small interfering RNA (siRNA) or antisense oligonucleotides (ASOs) to target the TTR protein in the liver before translation [9]. These therapies have already been in clinical use for familial amyloid neuropathy (FAP), given prior studies showing effectiveness in halting progression, or, in some cases, even reversing the neuropathy seen in these patients. The effectiveness of these agents was noted to be significantly higher than the levels seen in trials of TTR-stabilizing agents in FAP. This has led to significant interest in exploring their use in ATTR-CM.

#### 3.2.1. Patisiran

Patisiran is a siRNA encapsulated in a lipid nanoparticle, which allows for its uptake by the liver. It is FDA-approved for the treatment of hereditary ATTR-polyneuropathy, as the results from the phase 3 APOLLO-A trial demonstrated its ability to slow or reverse the progression of neuropathy and improve quality of life [18,19]. The siRNA encapsulated within lipid nanoparticles (LNP-siRNA) is designed to target the 3′ untranslated region of TTR mRNA in the liver to reduce translated TTR protein in both the ATTRm and ATTRwt [19]. The APOLLO-B trial was a multi-center, phase 3, double-blind, randomized, placebo-controlled trial of patisiran in patients with wildtype and hereditary ATTR-CM. Patients were randomized 1:1 into a treatment group that received patisiran, and a control group that did not receive it. Furthermore, the groups were further stratified based on factors such as race, treatment with tafamidis, and NYHA class [19]. The primary endpoint was change in functional capacity, which was measured by change in the 6 min walk test compared to baseline. Multiple secondary endpoints were measured as well, including KCCQ-OS score and composite scores of death from any cause, cardiovascular events, hospitalizations for any cause, and urgent visits for heart failure. Laboratory values such as NT-proBNP, troponin, and serum TTR were measured and compared to baseline over a 12-month period [19].

At 12 months, the patisiran group demonstrated a smaller reduction in the 6 min walk test. KCCQ-OS scores were slightly improved in the treatment group, while a decline was seen in the control group. This trial demonstrated that patisiran effectively preserved functional capacity, health status, and quality of life in patients with ATTR-CM, with the results extending over 12 months. These results align with prior research demonstrating the benefits of reducing circulating TTR levels in patients with ATTR polyneuropathy phenotype [19]. Safety outcomes were comparable between the patisiran and placebo groups, with most adverse events being mild or moderate, such as arthralgia secondary to drug instillation. Maurer et al. acknowledged the limitations of the trial, such as its inability to assess mortality or hospitalization outcomes due to the shorter 12-month follow-up duration, and the exclusion of patients with severe ATTR-CM [19]. As a consequence, the US FDA declined to approve patisiran for ATTR-CM in 2023, citing a lack of clear data demonstrating improvement in meaningful clinical outcomes and need for studies with longer follow-up durations. As a consequence, the drug’s maker, Alnylam, decided to pivot future research efforts toward newer generation silencer agents such as vutisiran. Patisiran, however, remains available for use in patients with hereditary ATTR-related polyneuropathy (hATTR-PN).

#### 3.2.2. Vutrisiran

Vutrisiran is a second-generation RNA interference agent that works to inhibit the hepatic synthesis of both ATTRwt and ATTRm [19]. Vutrisiran demonstrates greater potency than prior generations of TTR silencers through an N-acetylgalactosamine ligand that binds to hepatic asialoglycoprotein receptors. This increased potency allows for administration of the drug once every 3 months [20]. Similar to the APOLLO-A trial, the HELIOS-A trial demonstrated vutrisiran’s efficacy for ATTR patients with polyneuropathy. Based on those positive results, the HELIOS-B trial was started to investigate its efficacy in ATTR-CM.

The treatment group was given 25 mg of vutrisiran subcutaneously every 12 weeks for 36 weeks. The primary endpoint was a composite of death from any cause and recurrent cardiovascular events, such as hospitalizations for cardiovascular causes or urgent visits for heart failure, with secondary endpoints including functional capacity, quality of life, and cardiac biomarkers. The results demonstrated that vutrisiran significantly reduced the risk of the primary endpoint compared to placebo [20]. Vutrisiran significantly reduced mortality, with a hazard ratio in the overall population of 0.72 (95% confidence interval [CI], 0.56 to 0.93; *p* = 0.01) and a hazard ratio in the monotherapy population of 0.67 (95% CI, 0.49 to 0.93; *p* = 0.02) [19]. Additionally, vutrisiran better preserved functional capacity and quality of life, relative to the placebo group. No safety concerns arose during the trial, as the treatment and placebo groups endorsed similar rates of mild to moderate adverse side effects. Intriguingly, in a subgroup analysis, patients who were on tafamidis therapy at the time of enrollment in the trial benefited equally, with the addition of vutisiran, with those who were not on tafamidis. This suggests a possible benefit of the add-on therapy. However given the nature of the small numbers of this subgroup analysis, this will need to be explored further in larger studies [20].

Overall, the HELIOS-B trial strongly supports vutrisiran as an effective ATTR-CM therapeutic, and, in March of 2025, the FDA approved the drug for its use in ATTR-CM (both variant and wildtype) to reduce cardiovascular death, hospitalizations, and urgent heart-failure visits. Furthermore, amongst currently available agents for the use of TTR amyloid disease, it is the only therapeutic that has a dual indication for both neuropathy and cardiomyopathy, which are found concomitantly in many, if not most, hereditary or variant TTR patients.

#### 3.2.3. Eplontersen

Eplontersen is a ligand-conjugated antisense oligonucleotide directed to TTR, and it currently is under study in the CARDIO-TTRansform clinical trial for the treatment of ATTR-CM [21]. Eplontersen is targeted to the liver, where it then binds to wildtype and variant TTR mRNA to reduce the circulating levels of TTR protein. The drug is administered subcutaneously, and was shown to improve the health-related quality of life in patients with polyneuropathy of hereditary ATTR in a phase 3 clinical trial called the NEURO-TTRansform [21]. It has been approved for use in variant ATTR polyneuropathy; however, the utility of eplontersen in treating ATTR likely extends beyond polyneuropathy.

The CARDIO-TTRansform trial is currently investigating the efficacy and safety of eplontersen in patients with ATTR-CM [22]. CARDIO-TTRansform is a multicenter, double-blind study employing about 1400 participants. Participants will be randomized to receive subcutaneous injections of either eplontersen or placebo once every 4 weeks. The inclusion criteria state that the participants, either male or female and from the ages of 18–90, have amyloid deposits in cardiac or non-cardiac tissue, as confirmed with Congo Red staining or technetium scintigraphy. Furthermore, end-diastolic interventricular septum thickness must be >12 mm on an echocardiogram and the disease must have an NYHA class of I-III. Full exclusion criteria can be found at the CARDIO-TTRansfrom website (NCT04136171, https://clinicaltrials.gov/ct2/show/NCT04136171 (accessed on 10 February 2025). The study aims to measure a composite outcome of cardiovascular mortality and recurrent cardiovascular clinical events up to 140 weeks as a primary measure. Secondary outcome measures will comprise changes in the 6 min walk test distance and KCCQ scores, both at 121 weeks, and cardiovascular clinical events, mortality, and all-cause mortality up to 140 weeks [22]. The study is currently ongoing, and it is estimated that it will be completed in April 2026.

### 3.3. Transthyretin Gene Editing

Finally, CRISPR-Cas9-based gene editing offers the potential for a one-dose durable gene-silencing approach to ATTR-CM. In a pivotal pilot study by Gillmore et al., NTLA-2001 (also known as nexiguran ziclumeran), a CRISPR-Cas9 therapy targeting the TTR gene, was administered via a lipid nanoparticle (LNP) delivery system designed to edit TTR genes in hepatocytes, in six patients with hereditary TTR amyloidosis with polyneuropathy. This gene-editing system included mRNA encoding the Cas9 endonuclease and a single guide RNA (sgRNA) specific to the TTR gene. The goal of this therapy is to introduce frameshift mutations in the TTR gene, thereby preventing the production of both wildtype and mutant TTR, which are responsible for amyloid deposition [Figure 2] [23].

Following a single intravenous dose, NTLA-2001 demonstrated dose-dependent reductions in serum TTR levels. Patients receiving the 0.1 mg/kg dose experienced a mean reduction of 52% in serum TTR levels by day 28, while the 0.3 mg/kg group achieved an impressive mean reduction of 87%. These reductions were consistent across patients, ranging from 80% to 96% in the higher dose group. Preclinical studies further supported the durability of the edits, with TTR protein knockdown persisting over 12 months in animal models. Importantly, NTLA-2001 showed a favorable safety profile, with only mild, transient adverse events observed in the study cohort.

In a follow-up phase 1 study involving 36 patients, a single intravenous dose of nex-z led to rapid, deep, and durable reductions in serum TTR levels, with a mean reduction of 90% at 12 months. This significant decrease was accompanied by stability or improvement in clinical markers such as the 6 min walk test and NYHA class, suggesting a favorable impact on disease progression. The therapy was generally well-tolerated, with most adverse events being mild or moderate, including transient infusion-related reactions and liver enzyme elevations [24].

CRISPR-Cas9 gene editing marks a significant departure from the traditional therapies for ATTR-CM, including the TTR stabilizers and RNA-based silencing agents. Unlike these treatments, which require long-term administration, NTLA-2001 offers the potential for a single-dose, permanent solution to halt TTR production at its source. By targeting the genetic basis of ATTR, this approach could provide a more sustained control of amyloid deposition and reduce the burden of chronic therapy. Additionally, gene editing eliminates the challenges associated with adherence to lifelong treatment and the potential toxicities of repetitive dosing.

Despite these promising early results, this is an evolving field, with many challenges still to be addressed. The phase 1 trial enrolled a small number of patients with hereditary ATTR amyloidosis with polyneuropathy, leaving questions about its efficacy and safety in larger, more diverse populations, including those with cardiac involvement. Long-term follow-up is required to assess the durability of gene editing, potential off-target effects, and unforeseen complications such as immune responses to Cas9 or sgRNA components. Moreover, while the targeted nature of CRISPR-Cas9 minimizes off-target effects, rigorous genomic monitoring remains essential to ensure safety.

NTLA-2001 represents a paradigm shift in the treatment of ATTR-CM. By addressing the disease at its genetic root, this CRISPR-Cas9-based therapy could redefine the therapeutic landscape of a disease for which, until recently, treatments relied solely on stabilizers and silencers. As clinical trials expand, this approach may pave the way for broader applications of in vivo gene editing in cardiology and beyond.

## 4. TTR Scavenging Therapy

ATTR-CM has classically been associated with a poor prognosis, with current therapeutics offering prevention of disease progression but, thus far, no evidence of true reversal of disease. However, Fontana et al. described three male patients aged 68, 82, and 76 who exhibited near-reversal of their heart failure, with return to normal cardiac function [25]. All three patients were clinically similar in that they were not on any disease-modifying treatments, had no recent illnesses or vaccinations, and had similar cardiac findings on echocardiography and scintigraphy [25]. An endomyocardial biopsy was conducted on one patient, which revealed macrophages and giant cells surrounding the amyloid deposits. This prompted the authors to review previous biopsies conducted on other ATTR-CM patients, and none of them had similar findings [25]. All three patients had a large IgG titer against TTR amyloid, a determination which was absent in previous amyloid patients. These findings suggest a possible anti-amyloid immune mechanism that may challenge the notion that ATTR-CM is irreversible, and offer hope for a novel monoclonal antibody treatment.

While most therapies focus on targeting the TTR protein directly, monoclonal antibody treatment works to target pathologic TTR protein, while sparing physiologic protein [26]. The proposed mechanism is that monoclonal antibodies could be designed to target aggregated TTR protein in the myocardium via macrophages [Figure 2] [27]. Work on this approach has led to the rapid development of such antibodies, now in clinical trials.

One such antibody, ALXN2220 (also known as NI006 or NI301A), was engineered to have high affinity binding to ATTR, amyloid-removal activity, and lack of binding to naturally folded, physiological TTR [27]. ALXN2220 was found to bind to amyloid deposits in myocardium obtained from patient post-mortem tissue ex vivo as well as wildtype mice grafted with patient-derived amyloid fibrils [27]. These findings suggest that ALXN2220 could be a promising therapeutic candidate for reducing cardiac amyloid burden in patients with ATTR-CM. A phase 1, double blind trial randomly assigned 40 patients with either ATTRwt or ATTRm and chronic heart failure to receive either intravenous infusions of ALXN2220 or placebo every 4 weeks for 4 months. The treatment group received stepwise ascending doses of ALXN2220, from 0.3 to 60 mg per kilogram of body weight. Cardiac imaging, such as cardiac MRI and scintigraphy, primarily measured ATTR-CM regression, as did laboratory values such as NT-proBNP and troponin T levels [28]. ALXN2220 demonstrated a favorable safety profile, with no drug-related serious adverse events or antidrug antibody formation. At doses of 10 mg/kg or higher, reductions in extracellular volume on cardiac MRI and decreases in cardiac tracer uptake on scintigraphy were observed over 12 months [28]. Additionally, decreases in NT-proBNP and troponin T suggested improvements in cardiac function [28]. Adverse events, primarily mild musculoskeletal complaints and transient thrombocytopenia, were manageable. These results support the safety profile of ALXN2220 and highlight its efficacy in decreasing cardiac amyloid disease, demonstrating its potential to become a novel therapeutic, pending further clinical trial results. This antibody was designated for fast-track review, and enrollment for a phase 3 clinical trial, DepleTTR-CM, has already been completed, with study completion estimated to be sometime in 2027.

Another potential antibody scavenger therapy for ATTR-CM is PRX004, also known as coramitug. Similar to ALXN2220, PRX004 is an investigational humanized monoclonal antibody designed to specifically target and clear misfolded TTR amyloid deposits. It is currently being tested in a phase 1 open-label study of intravenous PRX004 as a single agent in subjects with hereditary amyloidosis. Dose-escalation studies demonstrated that PRX004 was well-tolerated at doses up to 30 mg/kg administered every 28 days, with signs of clinical efficacy including improved markers of heart and nerve function. A phase 2 randomized control trial has recently been completed, with results pending, and a phase 3 trial has been announced that will commence in 2025 [29].

## 5. Management of Cardiovascular Complications

While addressing the underlying pathology of ATTR-CM is essential, management of cardiovascular manifestations also remains a hallmark of therapy. The deposition and infiltration of amyloid fibrils leads to left ventricular wall thickening, while the left ventricular cavity remains at normal size. This restrictive cardiomyopathy can resemble HFpEF clinically, but can have distinct differences in clinical management. For instance, the conventional use of beta blockers, angiotensin-converting enzyme blockers, and angiotensin II receptors blockers may lead to potentiated hypotension as a side effect due to autonomic dysfunction related to amyloid autonomic neuropathy [7]. Furthermore, calcium channel blockers such as verapamil may cause potentiated negative inotropic effects by binding to highly concentrated areas of amyloid fibrils [6]. However, diuretics, mineralocorticoid antagonists, and sodium–glucose cotransporter 2 (SGLT2) inhibitors have all been found to be well tolerated, and their use is likely beneficial in the management of patients with ATTR-CM. Arrhythmia management can include pacemaker or implantable cardioverter defibrillator (ICD) implantation for those with bradyarrhythmias or tachyarrhythmias, respectively. One proposed approach to the management of these patients is CHAD-STOP: Conduction and rhythm disorders prevention, High heart-rate maintenance, Anticoagulation, Diuretic agents, and STOP ß-receptor and calcium-channel blockers, digoxin, and RAAS inhibitors [30].

## 6. Conclusions

ATTR-CM is a complex and progressive condition that demands a multifaceted approach to its management. Recent advancements in diagnosis, therapeutics, and emerging technologies have significantly expanded the arsenal of treatments available to cardiologists, offering new hope for improving patient outcomes. Traditional therapies such as symptom-targeted management and pacemaker implantation remain foundational but are increasingly complemented by disease-specific approaches, including TTR stabilizers and silencers and cutting-edge gene-editing techniques.

At this time, we are now fortunate to have a multitude of therapies to choose from for treatment of those afflicted with TTR amyloidosis with cardiac involvement, including both stabilizers and silencer therapies, both of which are approved in the USA. All three currently available therapies (tafamidis, acoramidis, and vutisiran) are associated with robust data supporting improvement in survival, reduced hospitalizations, and preserved functional capacity [Table 1]. Tafamidis, as the first agent in the stabilizer class, has shown excellent efficacy with very few adverse events in the real-world long-term data now extending 5 years and beyond. As previously noted, acoramidis, as a newer agent in this class, has demonstrated superior biochemical stabilization in vitro with excellent outcome data in its phase 3 trial. Clinical data regarding comparable efficacy between stabilizers is still lacking. What does seem to be clear, though, is that the concomitant use of dual stabilizer therapies does not improve outcomes. Silencing agents like patisiran, vutrisiran, and eplontersen provide promising alternatives, or possibly add-on therapies, for ATTR-CM, especially those cases with familial disease and concomitant neuropathy. At this time, only vutisiran has been approved in the USA for the treatment of cardiac disease, and it may be the initial treatment of choice in cases with both cardiomyopathy and neuropathy, given its dual indications.

Emerging therapies, including monoclonal antibodies such as NI006 and gene-editing platforms like NTLA-2001, signal a paradigm shift toward targeted amyloid clearance and potential curative approaches. While these innovations are still in the early stages of clinical development, they offer compelling avenues for addressing the underlying pathology of ATTR-CM.

Looking ahead, the challenge lies in integrating these novel therapies into clinical practice, tailoring treatment strategies to individual patient profiles, and addressing issues such as accessibility, cost-effectiveness, and long-term safety. For cardiologists, the growing body of evidence underscores the importance of early diagnosis and a multidisciplinary approach to optimize outcomes. As research continues to evolve, the future of ATTR-CM management holds promise for transforming what was once a devastating disease into a more manageable condition with durable outcomes.

## Figures and Tables

**Table 1:** Landmark trials and therapeutics.

Trial Name	Drug Studied	Mechanism of Action	Baseline Characteristics (Treatment Group)	Key Results	Survival at 30 Months
**ATTR-ACT** *NEJM, 2018*	Tafamidis	TTR stabilizer	91.3% Men 79.9% White Median age: 75 years Median NT-proBNP (pg/mL): 2995.9 NYHA II symptoms: 61.4% NYHA III symptoms: 29.5%	441 patients enrolled 267 → Tafamidis 177 → Placebo 30% reduction in all-cause mortality (29.5% in tafamidis group vs. 42.9% in placebo group, HR 0.70; 95% CI 0.51 to 0.96) 32% reduction in cardiovascular-related hospitalizations (0.48 per year vs. 0.70 per year; RR 0.68, 95% CI, 0.56 to 0.81)	Pooled Tafamidis: 70.5% Placebo: 57.1% RRR: 32%
**ATTRibute-CM** *NEJM, 2024*	Acoramidis	TTR stabilizer	91.2% Men 87.4% White Median age: 77 years Median NT-pro BNP (pg/mL): 2326 NYHA II symptoms: 69.6% NYHA III symptoms: 18.3%	632 patients enrolled 421 → Acoramidis 211 → Placebo Four-step primary hierarchical analysis (includes death from any cause, CV-related hospitalization, change from baseline in NT-proBNP level, and change from baseline in 6 MWD) yielded a win ratio of 1.8 (95% CI, 1.4 to 2.2) in acoramidis group vs. placebo	Acoramidis: 80.7% Placebo: 74.3% RRR: 25%
**APOLLO-B** *NEJM, 2023*	Patisiran	RNA interference (RNAi) therapeutic	89% Men 77% White Median age: 76 years Median NT-pro BNP (pg/mL): 2008 NYHA II symptoms: 86% NYHA III symptoms: 8%	360 patients enrolled 181 → Patisiran 179 → Placebo At 12 months, magnitude of decline in 6 MWD was significantly lower in the treatment group (median change from baseline of −8.15 m (95% confidence interval [CI], −16.42 to 1.50) in the patisiran group and −21.35 m (95% CI, −34.05 to −7.52) in the placebo group	30-month data N/A 12-month follow-up period did not provide sufficient power to assess the effects of treatment on end points related to mortality and hospitalization
**HELIOS-B** *NEJM, 2024*	Vutrisiran	RNAi therapeutic	92% Men 85% White Median age: 77 years Median NT-pro BNP (pg/mL): 2021 NYHA II symptoms: 77% NYHA III symptoms: 8%	655 patients enrolled 326 → Vutrisiran 329 → Placebo Approximately 60% of both groups not on tafamidis (monotherapy group) Vutrisiran showed a significant reduction in mortality, with a HR of 0.72; 95% CI, 0.56 to 0.93; *p* = 0.01, and a HR of 0.67 in the monotherapy population, 95% CI, 0.49 to 0.93; *p* = 0.02	Vutrisiran: 84% Placebo: 79% RRR: 24%
**CARDIO-TTRansform**	Eplontersen	Antisense oligonucleotide	Inclusion Criteria: Confirmed diagnosis of ATTR-CM End-diastolic interventricular septum thickness of >12 mm (mm) New York Heart Association (NYHA) class I-III	Trial is ongoing; results are pending.	N/A
**DepleTTR-CM**	ALXN2220	Monoclonal antibody against misfolded TTR	Inclusion criteria: Confirmed diagnosis of ATTR-CM End-diastolic septal wall thickness > 11 mm for women or >12 mm for men NT-proBNP > 2000 pg/mL Treatment with loop diuretic 30 days prior to screening NYHA class II-IV Life expectancy of >6 months	Trial is ongoing: results are pending	N/A

## Data Availability

Not applicable.
